# Unpacking language teacher beliefs, agency, and resilience in the complex, unprecedented time: A mixed-method study

**DOI:** 10.3389/fpsyg.2022.958003

**Published:** 2022-08-24

**Authors:** Yang Gao, Lili Qin, Qiyi Gu

**Affiliations:** ^1^School of Foreign Studies, Xi’an Jiaotong University, Xi’an, China; ^2^Department of World Languages, Dalian University of Foreign Languages, Dalian, China; ^3^School of Foreign Studies, Jiangnan University, Wuxi, China

**Keywords:** agency, resilience, language teacher beliefs, a mixed-method study, sociocultural theory (SCT)

## Abstract

We conducted this mixed-method study by focusing on the influx relationship among teacher beliefs, agency, and resilience during the pandemic and exploring the relationships and tensions among these constructs or capacities. Specifically, we surveyed 93 language teachers across seven different regions in China and collected their perceptions and beliefs about challenges and solutions during the first wave of the pandemic. In a further step, we interviewed six participants, analyzed the transcripts of the interviews, and then explored how their agency and resilience emerged and developed during the pandemic. From the quantitative statistics, we reported teacher beliefs about emotional, physical, mentoring, and support challenges in emergency remote teaching and their adopted strategies to handle these challenges during the pandemic. We also reported significant correlations among different perceived challenges and solutions. From the qualitative analysis, we found that language teacher beliefs, agency, and resilience co-evolved from intrapersonal and interpersonal reflections through temporal and contextual affordances. Drawing from the sociocultural theory, we contributed a theoretical framework for studying language teacher beliefs, agency, and resilience. We discussed our findings around the global traits that language teachers are required to develop in the increasingly complex world and also offered implications for language teacher education programs.

## Introduction

We live in a complex world. What makes it even worse is the outbreak of unexpected events or pandemics; therefore, we as teacher educators shoulder the responsibility to provide teachers with effective and efficient remedies that serve practical or psychological purposes during any emergency. A witnessed increase in the literature that helps teachers handle their emergency remote teaching (ERT) during the COVID-19 pandemic thus emerges. Most of these works fall into two tracks: some focus on the effectiveness of the instructional design and teaching strategies (e.g., Hodges et al., 2020), and the others focus on exploring how educational policies inform teachers’ planning and actual practices (e.g., Gao, 2020; Gao et al., 2022).

Specifically, studies revealed inconsistent findings on the effects of e-learning or online teaching, not typically for the ERT. Some confirmed the online teaching benefits, including real-time communication and interaction between teachers and students and advanced development of educational or instructional tools (e.g., Samir et al., 2014). However, other studies reported the drawbacks or challenges of e-learning, arguing that additional technological or operational knowledge derived from online teaching burdens teachers (e.g., Hodges et al., 2020). While the findings on the effectiveness of online teaching remain in a quandary, it is widely accepted that ERT facilitates instruction during unpredictable times (Gao et al., 2022). In addition, educational planning and policies helped teachers tackle demands and changes during the pandemic. While teachers’ beliefs and practices generally echoed these educational planning and policies, certain tensions between teachers’ beliefs and actual practices still existed (Gao et al., 2021, 2022).

With the proliferation of studies on ERT effectiveness and teacher beliefs about ERT, there is still a dearth of literature on how teachers’ beliefs, agency, and resilience co-evolve to help them conquer the demanding time. These teacher psychological constructs are, in nature, internal factors that fundamentally help teachers internalize ways or wisdom to handle challenges along their academic trajectory instead of randomly or blindly seeking external help from experts or peers. Extending this line of inquiry thus helps teachers develop professionally and practically during the pandemic. Some literature, through the increasingly promoted *positive psychology*, has advocated the need to study these psychological constructs over recent years. For example, in a conceptual piece, Wang et al. (2021) analyzed how seven psychological constructs, including engagement, emotion regulation, enjoyment, grit, loving pedagogy, resilience, and well-being, may contribute to desirable language learning and teaching experiences. Also, in another opinion paper, García-Álvarez et al. (2021) specifically focused on teacher well-being and proposed certain ideas and possible interventions to maintain and promote teachers’ well-being from the pandemic and adversity. Mercer and Gregersen (2020) also contributed to study teacher well-being from *positive psychology* and offered a multitude of approaches and strategies that teachers may reply on and practice to promote their own well-being, while some of these tenets may also find their root in previous works (e.g., Williams et al., 2015). However, there remains a scarcity of empirical studies or mixed-methods studies to show how teachers actually believe, act, and respond to the pandemic through these psychological constructs and their practice. Therefore, in this current study, we aim to address such a gap by exploring how teachers’ beliefs help them regulate agency and resilience and smooth over instructional difficulties during the pandemic.

## Literature review

### Language teacher beliefs, agency, and resilience

*Teacher beliefs* is a key concept in studying teacher practices and professional development, typically in disaster times. It has been extensively studied over the decades from multiple perspectives, including cognitive orientation (Borg, 2003, 2006, 2009, 2011), sociocultural theory (SCT) (Johnson, 1994, 2009), reflective practices (Farrell, 2013), and complex dynamic systems theory (Zheng, 2015; Gao, 2021a). It has also been studied from methodological approaches (e.g., Kalaja and Barcelos, 2003; Borg, 2012), review or historical analysis (e.g., Gao, 2014; Fives et al., 2019), and tensions between beliefs and practice (Borg, 2018; Gao and Bintz, 2019; Gao, 2021a). Mapping out the existing literature on teacher beliefs over the decades, Gao (2021a) explained that teacher beliefs are in nature complex, non-linear, and unpredictable and may include different theoretical orientations of teachers’ subject matter, matrixing in different forms to inform teachers of their practices. Teacher belief studies have shifted from a positive paradigm, exploring linear relationships between specific language skills including reading, writing, or grammar in particular (Farrell, 1999; Farrell and Lim, 2005; Farrell and Bennis, 2013; Zhang and Sun, 2022), to a pragmatism paradigm which highlights the complex tensions among constructs, including teacher identity and agency other than language skills (Mercer, 2012; Gao, 2014, 2021b; Borg and Sanchez, 2020; Golombek and Johnson, 2021).

Teacher beliefs connect teacher agency in a complex manner (Zhang and Liu, 2014; Zheng, 2015; Borg and Sanchez, 2020; Gao, 2021a). The two constructs often contribute in a collective way to enact specific educational programs. For example, Bonner et al. (2020) examined the cases of three schools with multiple years of experience implementing a STEM reform and related teachers’ experiences to their beliefs, goals, and plans as evolving agents in their school and the reform initiative. Teachers’ experiences, beliefs, and agency helped teachers implement, enact, and reform the program. In addition, teachers’ former experiences act as stimuli to guide teachers to perceive, analyze, and interpret dynamics in these issued programs and policies; their newly formed beliefs then inform teachers to act through the programs and policies, enhancing their agency in one way or another (Biesta et al., 2015; Kalaja et al., 2015; Agnoletto et al., 2021; Gao et al., 2022). Gao et al. (2022) metaphorically compared the educational planning process for ERT as a planning, implementation, and evaluation (PIE) and argued that baking the PIE required teacher agency. Biesta et al. (2015) also argued that sometimes teacher beliefs might be instrumental rather than altruistic or superficial rather than sophisticated, which makes teacher agency development lack a clear, robust professional vision. In other words, the construct of teacher beliefs itself may serve as the agent or starting point to develop teacher agency and support student learning or autonomy (Borg and Alshumaimeri, 2017; Borg and Parnham, 2020).

Similarly, resilience begins with teacher beliefs (Benard, 2004; Truebridge, 2014, 2016). Benard (2004) advocated that “we need to begin with belief in the innate resilience of every human being” (p. 113). Truebridge (2014) offered more than 150 examples of resilience in practice in classrooms, schools, and districts and argued that teacher beliefs that truly influenced these resilience in practice. Resilience relates to the presence of three interrelated protective factors including *caring relationships*, *high expectations*, and *meaningful opportunities* for participation and contribution (Benard, 2004, 2007). Fostering these three protective factors requires teachers’ powerful mindsets and beliefs (Truebridge, 2016).

In addition, teacher agency and resilience have been conceptualized from multiple perspectives, among which the social-ecological perspective is one dominant theoretical orientation (Priestley et al., 2015). The social-ecological perspective argues teacher agency is not a capacity or ability individuals inherit but instead a temporal and relational phenomenon emerging through interactions within ecological affordances (Van Lier, 2000; Biesta and Tedder, 2007). The agency development is thus viewed as an influx of influences and affordances, guiding teachers to develop on a scalar orientation, from the past to the present and then to the future (Priestley et al., 2015).

Similar to agency, resilience is also context-sensitive and role-specific, which requires teachers’ commitment and agency in the everyday world (Gu and Day, 2013). Agency and resilience, the two constructs, often contribute in a collective way to form and re-form teacher identity (Day, 2018). While the social-ecological perspective offers insights to study teacher beliefs, agency, and resilience as a system, it focuses more on the interaction between individuals and environments than on the internalization of the individuals. As these three constructs are psychological, a fit theoretical framework that helps explore how they evolve to the internalizing process of individual teachers may thus be required for the present study. Therefore, we chose SCT as the theoretical framework.

### Sociocultural theory as theoretical framework

As stated in the extensive literature, SCT approaches to language teacher beliefs and cognition focus on the individuals acting in a sociocultural setting (Johnson, 2018, 2019; Johnson and Golombek, 2020). Agency in SCT approaches is thus not simply defined as a voluntary control over one’s behavior but a relationship being co-constructed and co-negotiated with others in a social setting (Lantolf and Thorne, 2006), or “a contextually enacted way of being in the world” (Van Lier, 2008, p. 163). SCT approaches to study agency and resilience highlight the idea of mediation (Vygotsky, 1978) or mediation of learning experiences (Feuerstein and Feuerstein, 1991), which argues the human agent is not directly involved with his or her environment but mediated by artifacts or individuals (Wertsch, 1991, 1994, 1998).

Under the SCT framework, resilience is also conceptualized as a capacity of an individual teacher to harness personal and contextual resources to navigate through challenges in the sociocultural setting (Beltman et al., 2011; Beltman, 2015). Therefore, the study aims at exploring how individual teachers have developed teacher beliefs, agency, and resilience through different mediators and affordances.

## Research methodology

### Research background and setting

The fierce COVID-19 has been widely spreading across the globe since the beginning of 2020. By May 30, 2022, the global statistics have reached 529 million confirmed cases and 6.29 million deaths. One of the severe consequences that COVID-19 has caused is the majority of schooling to move online. In response to the emergency, different institutes worldwide, with their teachers and staff, actively initiated different plans.

In the current study which has been conducted in China, we traced sampled institutes and teachers from the beginning of February 2020, when the government decided to close campuses all across the country, to late May 2020 in the first wave of the outbreak, prior to the emergence of the Delta virus. We then explored how these sampled teachers had perceived challenges and acted during the pandemic, typically in the first wave.

### Research sample and participants

We started collecting data from a large sample of teachers in the first stage of the study. We sent out about 140 surveys but ended up collecting 93 copies of responses. These sampled teachers were from different regions in China, including east, north, south, central, northeast, northwest, and southwest regions. While we made attempts to run through random sampling, we still collected most surveys from the northeast region, from which came two of our principal investigators.

In the second stage, we further interviewed six participants from the selected universities. The participants were purposefully selected according to certain criteria, including but not limited to teacher willingness to participate, teacher availability for interviews, and the manageability of the study. Specifically, we included five female participants and three male participants. All had been working in their respective sites for 6 to 20 years at the time the study was conducted. Their ages ranged from 32 to 46. Table 1 lists the biographical information of the six purposive participants.

**TABLE 1:** Biographical information of the participants.

Participant	Pseudonyms	Gender	Age	Years of teaching experience
A	L	F	32	6
B	W	F	39	14
C	G	M	38	14
D	Y	M	36	10
E	Q	F	46	20
F	Z	M	36	6

M, male; F, female.

### Research methods: An exploratory sequential mixed-methods design

We adopted an exploratory mixed-method design (Creswell and Plano Clark, 2007; Creswell and Creswell, 2018). When choosing our research method, we considered whether the design fits our research aim, purposes, and questions. The primary purpose of this study is two-fold: we first aim to analyze teachers’ perceptions and beliefs about challenges and solutions during the first wave of the pandemic; we then aim to explore how teacher beliefs, agency, and resilience have evolved during the pandemic.

We mapped out two stages for this study according to our design. For the first stage, we used quantitative analysis to report basic descriptive and correlation analyses of teachers’ perceived challenges and solutions. The second stage involved exploring teachers’ perceptions and the evolution of their agency and resilience during the first wave of the pandemic. We used a semi-structured survey to interview participants and then analyzed their interview transcripts. To make our study manageable, we included only six purposive participants from each of our selected sites.

### Research instruments

As the study included two stages of design, we used a survey as a research tool to solicit teachers’ beliefs about challenges and methods to smooth over the challenges during the pandemic (Appendix I). We mapped out the existing literature (e.g., Biesta et al., 2015; Mansfield et al., 2016; Mansfield, 2020) and framed the survey with four dimensions of challenges, including *teacher emotional resilience*, *teacher physical resilience*, *teacher mentoring resilience*, and *teacher support resilience*. Accordingly, we included items specifying sub-dimensions for each dimension in the survey. Also, at the end of each dimension, we included a multiple-choice item recruiting teachers’ beliefs about the solutions or ways to handle these specified challenges or resilience. We originally designed the survey in Chinese, as all our participants were Chinese teachers. However, we used a translated version of the survey when we collected and analyzed our data. Also, we sent out our survey through a survey management platform (Wen Juan Xing) which helped us collect and analyze the data. We had excluded biographical information items in the survey and tested the survey for its validity and reliability before we sent them out to the sample participants. We obtained high reliability and validity for our survey, with the overall Cronbach’s alpha coefficient being 0.910 and Kaiser–Meyer–Olkin (KMO) value being 0.837.

It is worth mentioning that resilience and agency are dynamic and progressive, requiring detailed observations and descriptions instead of statistical analyses; therefore, in the quantitative stage, we referred to challenges and methods simply as something measurable but may offer insights to study resilience and agency. In the qualitative stage, we used interviews to further solicit teachers’ beliefs during the pandemic, which may inform their agency and resilience development.

### Data collection and analysis

We collected and analyzed data according to the two stages scheduled in the study. In the first stage, we collected responses on teacher beliefs about challenges and solutions to these challenges during the pandemic. We then ran basic descriptive and correlation tests for the survey items through statistical software and reported the statistics and figures afterward.

In the second stage, we interviewed the six participants, transcribed the interviews, and then analyzed the transcripts. Using the items in the survey as the starting points or referents, we guided the teacher participants to provide rich information for their responses. In several cases, we returned to our interviewees or participants to further solicit answers to a handful of questions. By doing so, we made our coding systemic and our descriptions saturated.

Ethical considerations were seriously taken into consideration when this empirical study was conducted. Specifically, we delivered copies of consent forms to participants and informed them of the research objectives and the confidentiality of their answers. With the participants’ signed consent forms, we then guided the participants through the whole process. We also kept all the data and transcripts confidential and acknowledged the sample and participants’ efforts in making the study, typically for the data collection and analysis process, possible.

## Findings

### Quantitative descriptions: Teachers’ beliefs about challenges and solutions during the COVID-19

Table 2 presents quantitative descriptions of teachers’ beliefs about challenges and solutions during the COVID-19. Items 5 and 6 solicited teachers’ perception of and panic over the pandemic and their perception of the influence of the pandemic on their families. Statistically, 42.00% of the sample chose “average” for item 5, indicating that most teachers were not worried about the pandemic. In item 6, 40% of the sample chose “average,” also indicating that these teachers did not believe that the pandemic would affect their families. However, in item 7, which required teachers to report whether they believed the pandemic would affect their schooling and teaching, over 30% of the sample chose “fair,” indicating that the way of online courses posed some challenges for teachers. That is the same for item 8; over 30% of the sample chose “fair,” indicating that the assessment of teaching caused some degree of anxiety for teachers.

**TABLE 2:** Basic description of the teacher beliefs about challenges.

Item	Categories	Frequency	Percent (%)	Cumulated percent (%)
5. During the pandemic, did you feel panic and anxious? (Example: media coverage of the new coronavirus)	None	12	24	24
	Rarely	14	28	52
	Average	21	42	94
	Frequently	2	4	98
	Always	1	2	100
6. During the pandemic, were you worried about the health of your family and yourself?	None	8	16	16
	Rarely	12	24	40
	Average	20	40	80
	Frequently	5	10	90
	Always	5	10	100
7. Did emergency remote teaching (ERT) bring you more challenges and workload?	None	8	16	16
	Rarely	7	14	30
	Average	19	38	68
	Frequently	11	22	90
	Always	5	10	100
8. During ERT, were you worried and anxious about the evaluation of teaching performance?	None	10	20	20
	Rarely	14	28	48
	Average	19	38	86
	Frequently	5	10	96
	Always	2	4	100
9. Were you afraid of ERT because of your lack of online teaching experience?	None	24	48	48
	Rarely	15	30	78
	Average	10	20	98
	Frequently	1	2	100
	Always	0	0	100
11. Do you think the Internet, applications, or teaching management platforms have brought you challenges during the online teaching period?	None Rarely Average Frequently Always	22 31 33 5 2	24 33 36 5 2	24 57 92 98 100
12. During ERT, do you think the Internet, application software, or teaching management platform brought you challenges?	Rarely	6	12	12
	Average	9	18	30
	Frequently	17	34	64
	Always	10	20	84
	Always	8	16	100
13. Did your office location or electronic equipment make it difficult for you to teach online?	None	15	30	30
	Rarely	15	30	60
	Average	11	22	82
	Frequently	6	12	94
	Always	3	6	100
14. Did you feel physical discomfort during online classes?	None	20	40	40
	Rarely	11	22	62
	Average	13	26	88
	Frequently	4	8	96
	Always	2	4	100
16. Were you worried that students would have depression, paranoia, stress, and other negative emotions during online teaching?	None	10	20	20
	Rarely	5	10	30
	Average	20	40	70
	Frequently	12	24	94
	Always	3	6	100
17. Do you think the pandemic would affect the graduation or work situation of students?	None	3	6	6
	Rarely	4	8	14
	Average	23	46	60
	Frequently	13	26	86
	Always	7	14	100
18. Were you concerned about your students’ class attendance during ERT?	None	13	26	26
	Rarely	17	34	60
	Average	14	28	88
	Frequently	3	6	94
	Always	3	6	100
19. During ERT, were you concerned about students’ use of technology operations?	None	23	46	46
	Rarely	15	30	76
	Average	11	22	98
	Frequently	1	2	100
	Always	0	0	100
20. During ERT, were you concerned that students will not have a suitable location/electronic device to attend class?	None	16	32	32
	Rarely	16	32	64
	Average	14	28	92
	Frequently	4	8	100
	Always	0	0	100
21. During ERT, were you worried that students’ lack of online learning experience would reduce their learning efficiency?	None	6	12	12
	Rarely	16	32	44
	Average	12	24	68
	Frequently	10	20	88
	Always	6	12	100
22. Were you worried about the physical condition of your students during online teaching?	None	9	18	18
	Rarely	14	28	46
	Average	19	38	84
	Frequently	4	8	92
	Always	4	8	100
24. Did you encounter challenges from school or departmental systems while teaching online? (For example, the relevant regulations cannot be implemented in a timely and effective manner)	None	16	32	32
	Rarely	16	32	64
	Average	16	32	96
	Frequently	1	2	98
	Always	1	2	100
25. During ERT, did you experience challenges due to a lack of peer/expert support (e.g., no peer model or technologist for help)	None	19	38	38
	Rarely	19	38	76
	Average	10	20	96
	Frequently	2	4	100
	Always	0	0	100
26. During ERT, did you encounter challenges from the surrounding environment (such as unstable community network, frequent nucleic acid testing, etc.)	None	9	18	18
	Rarely	12	24	42
	Average	20	40	82
	Frequently	7	14	96
	Always	2	4	100
27. Did you encounter challenges with your child’s learning during online teaching? (For example, children need to be accompanied by parents, time conflicts, etc.)	None	20	40	40
	Rarely	8	16	56
	Average	9	18	74
	Frequently	12	24	98
	Always	1	2	100

In item 9, over 40% of the sample selected “no,” indicating that most teachers were not afraid of online teaching in spite of their lack of teaching experience, which means that online teaching did not affect the teachers very much. In item 12, 34% of the sample chose “average,” indicating that teachers did not find online teaching difficult for student–teacher interaction. In item 13, 30% chose “no,” indicating that office locations and electronic devices did not make online teaching challenging. In item 14, more than 40% of the participants did not find online teaching physically difficult or demanding. This result is similar to item 9, which means that the way of the online class did not influence the physical condition. In item 16, 20 subjects, or 40%, chose “average,” indicating that teachers did not believe online teaching affected students’ emotions too much. In item 17, 46% of the subjects chose “average,” meaning that most teachers believed the pandemic would impact graduation and employment. In item 18, 34% of the respondents chose “rarely,” indicating that teachers did not think online teaching significantly impacted attendance. For item 19, the majority of respondents selected “no” which indicates that teachers generally believed that students were proficient in using devices to listen to lectures online. For item 20, the percentages of “no” and “rarely” were 32%, respectively, which indicates that the majority of students had access to online classes.

In item 21, 32% of the respondents chose “rarely,” indicating that teachers did not believe that lack of experience with online learning reduced learning effectiveness. In item 22, more than 30% chose “average,” indicating that most teachers believe that online instruction hardly had a negative impact on student’s health. For item 24, the majority of respondents chose “no,” indicating that schools were well supported by teachers during the pandemic. For item 25, 38% selected “rarely,” indicating that the majority of teachers were supported by peers and experts for online instruction. For item 26, 40% of the respondents chose “fair,” indicating that the environment did not have a significant impact on online instruction. For item 27, 40% chose “no,” but 24% chose “often,” suggesting that children home schooling during the pandemic, for teachers with children, influenced teachers’ online instruction to some degree.

In terms of teacher perceptions of the solutions to conquer challenges during the pandemic (see Table 3), 60% of teachers believed that “comfort myself” was an effective measure, and 78% did not believe that effective help or support could be sought from friends, studies, or psychological institutions; half of the respondents said that support from family members could effectively relieve emotional orientation, and 68% reported that distraction, or getting rid of thinking about the pandemic, could effectively relieve anxiety. In terms of teacher instruction, 94% of respondents felt it was important to have a learning management platform or technical software from the school, more than half felt it was important to simplify the online course process, and 84% felt it was important to communicate with students and familiarize themselves with the online process in advance. In terms of support for students, 72% of teachers believed students should be given help and comfort, 82% believe students should be guided to put their minds at ease, 74% believe students should be given technical help, and only 22% believed students should be guided to reduce stress. In terms of support for teachers, when faced with any ERT issue, only 34% would choose to go to their peers or school for support at work, 78% would choose to give timely feedback on the problem to their school leaders, 90% would choose to communicate with community workers, and only 4% would solve the problem by rationalizing their work schedule.

**TABLE 3:** Basic description of the teacher beliefs about solutions.

Item	Categories	Frequency	Percent (%)	Cumulated percent (%)
**Teacher emotional resilience**				
Comfort myself	No	20	40	40
	Yes	30	60	100
Seek help from friends, schools, and mental institutions	No	36	72	72
	Yes	14	28	100
Seek help from family	No	26	52	52
	Yes	24	48	100
Distract attention	No	16	32	32
	Yes	34	68	100
Others	No	40	80	80
	Yes	10	20	100
**Teacher physical resilience**				
Universities provide the platform and expenditure	No	47	24	24
	Yes	14	28	52
Simplify the online course process	No	24	48	48
	Yes	26	52	100
Talk with students more frequently	No	8	16	16
	Yes	42	84	100
Familiarize with the online teaching process in advance	No	8	16	16
	Yes	42	84	100
Others	No	12	24	24
	Yes	14	28	52
**Teacher mentoring resilience**				
Give comfort to students	No	14	28	28
	Yes	36	72	100
Guide students to relax their mentality	No	9	18	36
	Yes	41	82	100
Provide technical support to students	No	37	74	74
	Yes	13	26	100
Guide students to reduce the burdens	No	11	22	22
	Yes	39	78	100
Others	No	50	100	100
	Yes	0	0	100
**Teacher support resilience**				
Seek help from peers and school	No	17	34	34
	Yes	33	66	100
Give feedback to the school	No	39	78	78
	Yes	11	22	100
Communicate with staff in the community	No	45	90	90
	Yes	5	10	100
Management for working hours	No	2	4	4
	Yes	48	96	100
Others	No	40	80	80
	Yes	10	20	100

In terms of teacher emotional resilience (see Table 4), we used correlation analysis to investigate the relationship between item 5 and items 6, 7, 8, and 9, respectively. The correlation coefficient value between item 5 and item 6 was 0.593 and showed significance at a 0.01 level, which indicates that there was a significant positive correlation between teachers’ anxiety during the pandemic and teachers’ concerns about their families’ health. The correlation coefficient between item 5 and item 7 was 0.243, which was close to zero, and the *p*-value was 0.088 > 0.05, thus indicating that there was no association between teachers’ own anxiety and the challenges posed by online teaching. The correlation coefficient value between item 5 and item 8 was 0.283 and showed significance at a 0.05 level. This indicates that there was a significant positive correlation between teachers’ own anxiety and the anxiety associated with teaching assessment. The correlation coefficient between item 5 and item 9 was 0.298 and showed a significant level of 0.05. This indicates that there was a significant positive correlation between teachers’ own anxiety and teaching anxiety due to lack of experience.

**TABLE 4:** Correlations between teachers’ beliefs about emotional challenges.

	Item 5
Item 6	0.593**
Item 7	0.243
Item 8	0.283*
Item 9	0.298*

*p < 0.05; **p < 0.01.

As for teachers’ physical resilience (see Table 5), we used correlation analysis to analyze the relationship between item 14 and item 11, item 12 and item 13, respectively. The correlation coefficient value between item 14 and item 11 was 0.568 and showed significance at the 0.01 level. This indicates that there was a link between physical discomfort caused by online instruction and the challenges posed by the Internet teaching platform. The correlation coefficient between item 14 and item 12 was 0.404 and showed a 0.01 level of significance. This indicates that there was a link between physical discomfort due to online instruction and the teacher–student interaction of online instruction. The correlation coefficient value between item 14 and item 13 was 0.444 and showed a 0.05 level of significance. This indicates that there was an association between physical discomfort due to online instruction and hardware facilities for online instruction.

**TABLE 5:** Correlations between teachers’ beliefs about physical challenges.

	Item 14
Item 11	0.568**
Item 12	0.404**
Item 13	0.344*

*p < 0.05; **p < 0.01.

As for teacher mentoring resilience (see Table 6), we used correlation analysis to analyze the relationship between item 16 and item 17, item 18, item 19, item 20, and item 21, respectively. The correlation coefficient value between item 16 and item 17 was 0.515 and showed significance at a 0.01 level, thus indicating a significant positive correlation between these two. The correlation coefficient value between item 16 and item 18 was 0.225 and showed a significance at a 0.05 level, thus indicating a significant positive correlation between these two items. The correlation coefficient value between item 16 and item 19 was 0.195, which was close to 0, and the *p*-value is 0.061 > 0.05, thus indicating that there is no correlation between these two items. The correlation coefficient value between item 16 and item 20 was 0.214 and showed a significance at a 0.05 level, thus indicating that there was a significant positive correlation between these two. The correlation coefficient value between item 16 and item 21 was 0.414 and showed a significance at the 0.01 level, thus indicating a significant positive relationship between these two.

**TABLE 6:** Correlations between teachers’ beliefs about teacher mentoring resilience.

	Item 16	Item 22
Item 17	0.515**	0.331**
Item 18	0.225*	0.182
Item 19	0.195	0.252*
Item 20	0.214*	0.425**
Item 21	0.414**	0.338**

*p < 0.05; **p < 0.01.

As for the relationship between teacher emotional and physical concerns in the mentoring resilience, we used correlation analysis to analyze the relationship between item 16 and item 22 (see Table 7). The correlation coefficient value between item 16 and item 22 was 0.486 and showed significance at the 0.01 level, thus indicating a significant positive correlation between item 16 and item 22.

**TABLE 7:** Correlations between teacher mentoring resilience: Emotional vs. physical concerns.

	Item 16
Item 22	0.486**

**p < 0.01.

We used correlation analysis to analyze the relationship between item 24 and item 25 and item 27, respectively (see Table 8). The correlation coefficient value between item 24 and item 25 was 0.503 and showed a significance at a 0.01 level, thus indicating that there was a significant positive correlation between these two. The correlation coefficient value between item 24 and item 27 was 0.122, which was close to 0, and the *p*-value is 0.244 > 0.05, thus indicating that there was no correlation between these two.

**TABLE 8:** Correlations between teachers’ beliefs about teacher support resilience.

	Item 24	Item 26
Item 25	0.503**	0.546**
Item 27	0.122	0.198

**p < 0.01.

In addition, we used correlation analysis to analyze the relationship between item 26 and item 25, and item 27, respectively (also see Table 8). The correlation coefficient value between item 26 and item 25 was 0.546 and showed a significance at the 0.01 level, thus indicating a significant positive correlation between these two. The correlation coefficient value between item 26 and item 27 was 0.198, which was close to 0, and the *p*-value was 0.057 > 0.05, thus indicating that there was no correlation between the two.

To sum up, we summarized the following quantitative findings: in terms of teacher emotional resilience, teachers’ overall anxiety due to the pandemic was strongly correlated with their concerns about family members’ safety. In addition, teachers’ overall anxiety was correlated with their lack of online teaching experiences and their anxiety about being assessed for their ERT performance. However, teacher anxiety from the pandemic was not correlated with their concerns about extra workload from ERT preparation.

In terms of teacher physical resilience, it was related to the graduation or work situation of students affected by the pandemic, students’ use of technology operations, students’ access to the suitable location/electronic device to attend class, as well as students’ lack of online learning experience. However, students’ class attendance in the online teaching did not affect teacher physical resilience.

As for teacher mentoring resilience, it was similar to the teacher physical resilience. Teacher mentoring resilience was also affected by the graduation or work situation of students in the COVID-19, students’ access to the suitable location/electronic device to attend class as well as students’ lack of online learning experience. In addition, it was also impacted by the student’s class attendance. However, it was not affected by the students’ use of technology operations. Regarding teacher support resilience, lack of peer/expert support had an influence on it.

Meanwhile, we generated the following findings in teacher solutions in handling resilience during the pandemic: in terms of dealing with teacher emotional resilience, most teachers chose to seek help from their friends, schools, and psychists (72%). In handling teacher physical resilience, teachers preferred simplifying their teaching process or steps (48%) to other ways to handle physical resilience. In terms of handling teacher mentoring resilience, most teachers (74%) believed providing their students with technical support in an effective way. In handling teacher support resilience, most of the teachers (98%) believed communicating properly with staff in their community may help release tensions in teacher support resilience. In addition, 78% of the surveyed teachers believed giving feedback to their school may also help smooth over issues or challenges in teacher support resilience.

### Qualitative analysis: Teacher beliefs, agency, and resilience during the pandemic

While we found the quantitative findings may provide the reader with a general picture of their perceived resilience and their reported solutions which were connected with the resilience and reflected their agency, we also found them unable to capture dynamics and nuances among our studied constructs, including teacher beliefs, teacher agency, and teacher resilience. Therefore, we further explored these dynamics and nuances and depicted the qualitative findings as follows:

#### Agency and resilience co-evolve through intrapersonal and interpersonal reflections

Teacher beliefs are in nature statable (Gao, 2021a), which paves the way to interpret and understand teachers’ development of agency and resilience. In the study, these interviewed participants all reported how they had perceived challenges in instructional and mentoring practices and also how they had managed to handle these challenges and made their lecturing or schooling possible. This entire process required efforts from the participant teachers to reflect and also project a trajectory through which their agency and resilience had co-evolved and developed, intrapersonally or interpersonally (Mercer, 2012).

Intrapersonally, agency, and resilience among different individual teachers varied in their emergence forms during the process. For example, Participants A and E both reported how they had suffered from panic and anxiety when the pandemic emerged in late 2019. However, their perceptions of the panic might stem from different sources. For example, Participant E was panic and anxious because she had to wait for a long time before she would get something certain about the upcoming semester. The time and uncertainty caused her pain for a long period.

**Excerpt 1:**

“Believe it or not, I was so panicked and shocked when the outbreak was reported right before the Chinese New Year. I was then spending time with my husband’s family in his hometown and then was advised to leave even before the new year. My husband was worried that if we left late, we would be locked down in his hometown. Ever since I came back home, I have been waiting for the notice or announcement from my university. It was a suffering and painful experience.” **(Interview with Ms. Q, the Participant E)**

Unlike Participant E, Participant C felt painful because of the projected extra workload instead of the long waiting or uncertainty; however, he took it for granted finally and adapted to the emergency quickly.

**Excerpt 2:**

“Luckily, I have tons of experiences learning and even teaching online when I was in the United Kingdom. I was thus not that panic when I got a general announcement from my affiliate informing us of planning online classes for the new semester. However, I was not in a great mood as I could imagine there might be more workload in scheduling and preparing these online classes. But I took it as something unavoidable and then re-adjusted my mood just within a few days.” **(Interview with Mr. G, the Participant C)**

While the development of agency takes time, it benefits individuals with an accumulation of perceived positive outcomes (Wray and Richmond, 2018). The way that individuals melted perceived challenges, strategies, implementation, and positive outcomes represents the process of developing individuals’ resilience. In the study, resilience and agency did co-evolve through the planning and preparing process. For example, Participant C was actually reluctant to take the online teaching mode, as he thought he would do something more than what he used to do for the face-to-face offline classes. However, agency emerged when he faced the reality that he had to meet the teaching load requirements and also his accountability as an instructor or teacher.

Similar to Participant C, Participants B and F also believed extra preparation work would deprive them of doing research which was part of their research load in the research-based universities. However, they differed in their solutions in handling this preparing work due to different policies from their affiliates. Participant B’s affiliate empowered them to do whatever was simple and convenient as long as they met the teaching load and completed their lectures online. However, Participant F’s department and school followed strict teaching evaluation criteria that would inevitably force their teaching staff to prepare their lectures and quizzes to the norms or criteria. While these teachers believed extra preparation load would be seen as a burden or challenge, they still managed to take the load and planned their lectures in the end.

Interpersonally, teachers’ agency and resilience developed through influences from their peers, colleagues, or friends, either actively or passively. For example, Participant D was stimulated by his colleague to plan his lectures and set up a role model for his colleagues in his team. With that stimulus and her accountability as a team leader or associate department director, he managed to prepare his lectures.

**Excerpt 3:**

“I was actually ‘passively’ motivated by my peer colleague. And he took the lead in organizing and planning for his classes and set up a good role model for all my colleagues and friends. Then, as the associate director for my department, I had to prepare my lectures well. While that was seen as kinda ‘involution,’ it indeed motivated me to do better than I was supposed to do.” **(Interview with Mr. Y, the Participant D)**

Different from Participant F, Participant B acknowledged she gained support from her colleagues, who made her planning of the online lectures easy and possible. Her adaption to online teaching became active with her peers’ support and help.

**Excerpt 4:**

“We were actually grateful to some of our colleagues and also the school. They demoed how we could plan and deliver our lectures through some advanced learning management systems, some of which were quite new to us. Thanks to their help, we finally made lectures online. Gradually, I began adjusting myself to this online teaching platform.” **(Interview with Ms. W, the Participant B)**

Mercer (2012) defined agency through sensual and behavioral dimensions: a sense of agency concerns how agentic an individual feels both generally and specifically in particular contexts, and an agentic behavior entails an individual choosing to exercise or practice. In the current study, the sense of agency is further aligned with teachers’ beliefs and interpretations of the challenges and difficulties of planning their online lectures, while the act of agency, or the agentic behavior, is connected with teachers’ actual practices in taking action and delivering the lectures. Agency and resilience worked in the connecting process through teacher activities and groups (Borg et al., 2020).

#### Agency and resilience co-develop through temporal and contextual affordances

“It is clear that one’s sense of agency and beliefs about appropriate agentic behavior stem from how one interprets past experiences” (Mercer, 2012, p. 49). In other words, teachers’ beliefs and interpretations of their past experiences may inform their agency and resilience development in a scalar approach, which leads teachers to reflect (Gao, 2020). Therefore, agency and resilience development can be seen as a continuum consisting of temporal but valuable moments, which represents a social-ecological perspective that regards teacher agency, not as a capacity or ability but as a temporal and relational phenomenon that emerges during interaction within ecological circumstances (Biesta and Tedder, 2007; Priestley et al., 2015; Xun et al., 2021).

**Excerpt 5:**

“Actually, I was not aware that the pandemic would be so serious, as I had never experienced such a pandemic; even for SARS, I was so young at that time and wasn’t able to recall how I had been feeling. I just thought it would be the pandemic lasting for a couple of days until I got an announcement from my school… Now, I regard this as a treasurable and painful experience for our generation and also my son’s generation. We will be there…” **(Interview with Mr. G, the Participant C)**

Similar to Participant D, Participant D regarded the experience as a life lesson and also reported her growth through the journey. In other words, she believed that people going through the pandemic might experience something different from those who have not experienced any disaster or pandemic. This different experience was, in nature, something causing people to make changes, choices, or even adaption, a process that required people’s agency and resilience and can thus be reviewed as a treasurable process (Goller and Paloniemi, 2017). Specifically, for teachers, they activated their agency, focused on student learning to make choices, and took agentic actions to maintain the effectiveness of their teaching by battling with the challenges during the pandemic (Xun et al., 2021).

Teacher agency and resilience were thus not only momentary or temporal but also dynamic as they varied across time. Drawing from an ecological perspective, Biesta and Tedder (2007) interpreted the achievement of agency as a configuration of past influences, projections, or orientations toward the future, and engagement with the present, which represent an international, a projective, and an evaluative dimension, respectively (Priestley et al., 2015).

**Excerpt 6:**

“Yes, I was panic (at that time when the outbreak emerged), as I really knew nothing about how we would conquer the pandemic period, but now as you ask me to recall that experience, I just think that was not something big. It was just a lesson that we learned.” **(Interview with Ms. S, the Participant B)**

Agency is also affected by the culture in which an individual works (Buchanan, 2015). In the study, teachers were not just reactive to context, they acted as complex human beings or agents to make sense of and engage with contexts. Through the sense-making and engaging process, their resilience emerged and developed. These contextual factors may be exerted from different sources, in familial, institutional, or whatever format.

For example, Participant B had to take care of her babies when the pandemic broke out. This maternal responsibility was bound to her familial context and made her reluctant to plan for her online lectures.

**Excerpt 7:**

“… I was so reluctant to even think about planning for the online lectures because I had to take care of my babies. You know, they are so little. When it finally came to the new semester, I still managed to complete my preparation; even though I was staying quite late the week before the new semester. after all, that was my job.” **(Interview with Ms. S, the Participant B)**

Institutional affordances may also be seen as a certain contextual factor that actives teachers’ agency and resilience. David (2018) argued: “to understand the role of agency in resilience is to understand how individuals and collective agents behave in relation to varying contingencies, underpinned by factors and institutional mechanisms by which decisions are made and adaptive capabilities, obtained” (p. 1046). In the study, Participant A received favorable affordances from her department, which fostered her agency in smoothing over challenges in the first semester during the pandemic.

**Excerpt 8:**

“In our department, we were in the first semester asked to do whatever we like and find convenient, but just ensured the instruction could be implemented smoothly. This saved us from technology or applications; some senior professors really could not handle too many platforms or apps at the same time, so they just grouped all the students through WeChat and even voiced these messages piece by piece. Anyway, we all met the basic requirements, and rest assured our lectures were delivered during the first semester of the pandemic.” **(Interview with Ms. F, the Participant A)**

As stated above, Participant A received favorable policies from her affiliate and was empowered to simplify the lecture planning or preparing process. This saved the teacher from spending time teaching and preparing for lectures and even suffering from mental stress due to the pandemic. While Participants A and B were both agents acting through perceived affordances, these affordances may appear in different forms, either as nourishment for Participant A or detergent for Participant B. These teachers filtered these affordances and acted through their resilience.

## Discussion

**FIGURE 1:**
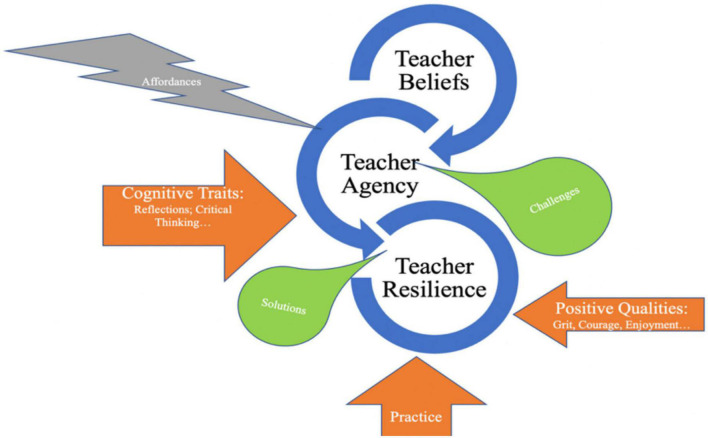
An SCT framework in studying teacher beliefs, agency, and resilience.

We presented our findings *horizontally* and *vertically* in the above section. Horizontally, we reported our quantitative data in terms of teacher emotional, physical, mentoring, and support resilience; we also reported teachers’ solutions in addressing ERT challenges during the pandemic. Vertically, we depicted how teacher beliefs, teacher agency, and teacher resilience co-evolved during the ERT period interpersonally and intrapersonally through temporal and contextual affordances. With the findings of the study, typically for the qualitative ones which depicted the dynamics and co-evolution of teacher beliefs, agency, and resilience, we would like to propose an SCT framework (Figure 1) for studying teacher beliefs, agency, and resilience, the three constructs or capacities that we have explored through the study.

An SCT approach to teacher education focuses only not on the interactions between individual teachers and their contexts or environments but also on how individuals’ senses and actions through these interactions can be turned into internalization of their life or professional career treasures (Johnson, 2009). Therefore, teacher beliefs serve as the starting point for teachers to activate their agency and then resilience, and they help teachers to interpret and understand their received information and then make their choices and decisions in analyzing the information. The analyzing process is part of the agency activation process, as agency stems from its sense level (Mercer, 2012). Teacher beliefs require teachers’ cognitive traits, including reflections and critical thinking to help teachers filter information and move the teachers through the decision-making process. Reflections and critical thinking help teachers extract important information from their past experiences or repertoire and filter important information to guide their practice. Emirbayer and Mische (1998) stated: “the selective reactivation by actors of past patterns of thought and action, routinely incorporated in practical activity, thereby giving stability and order to social universes and helping to sustain identities, interactions, and institutions over time” (p. 971). Gao (2021a) reported practice might be seen as both a product and also a premise for teacher beliefs. It is thus a go-between to mediate psychological constructs as teacher beliefs, agency, and resilience.

With the filtered information, the agency then activates its practical or behavioral level in further perceiving and interpreting challenges or difficulties and then providing the teacher agents with solutions and methods to handle these challenges or difficulties. Successfully smoothing over the challenges or difficulties requires teachers’ positive qualities, including grit, courage, and enjoyment. The existing literature provides the reader with conceptual pieces (Mercer et al., 2016) and empirical studies (Alrabai, 2022; Liu and Chu, 2022) that evidence positive emotions broaden people’s vision, enhance their strengths, and also alleviate negative emotions. Teachers’ positive qualities typically work in unprecedented time to enhance teacher agency and resilience.

It is also worth mentioning that affordances are perceived as an inseparable concept in the framework, as it provides teachers with premises, prerequisites, or even stimuli for activating agency and resilience. Van Lier (2004) argued that affordance might appear in different forms, including cultural affordances, social affordances, cognitive affordances, and so on, which may guide teachers to perceive and interpret challenges and practice with solutions in an authentic classroom.

## Conclusion

We enclose the paper by summarizing the implications and limitations of the study. With the findings and discussion of the study, we provide teacher educators with insights into training our preservice or in-service teachers: first, positive qualities serve a powerful role in shaping teachers’ beliefs, agency, and resilience. We may encounter challenges or even catastrophes on a regular basis in the professional trajectory. Keeping enough positive qualities in mind enables us to go further. While these positive qualities are essential for the development of teacher agency and resilience, they are actually companions of cognitive traits and behavioral practices from teachers. Reflective practices and critical thinking are constantly required through teachers’ resilience. Therefore, for teacher educators, how to provide preservice or in-service teachers with professional development programs highlighting the importance of positive psychological and cognitive traits becomes something indispensable. While we intend to provide teachers with insights to develop themselves either internally or practically during the pandemic, the pandemic itself made the data collection and analysis processes of the study challenge. These processes, which could have been done in an even more robust way than in its current version, could be seen as one of the limitations of the study. Another limitation would be the convenience sampling method, typically in the qualitative stage. Considering our nearly 100 participants in the quantitative stage, we were supposed to have more participants in the qualitative stage. However, due to the manageability challenges during the pandemic, we have already done our best in locating and following up with our participants in such a size. We are hoping that similar studies addressing these limitations will be conducted in the future.

## Data availability statement

The raw data supporting the conclusions of this article will be made available by the authors, without undue reservation.

## Ethics statement

Ethical review and approval was not required for the study on human participants in accordance with the local legislation and institutional requirements. The patients/participants provided their written informed consent to participate in this study.

## Author contributions

YG contributed to the overall design, organization, and logic of the manuscript. LQ and QG have contributed to the organization, design, and implementation of the study. All authors have contributed to the data collection, analysis of the manuscript, and drafting, revising, and proofreading of the manuscript, and approved the submitted version.

## Appendix I

Serial number Time to submit the answer sheet

The time spent Source

**Survey on Chinese college language teachers’ beliefs about agency and resilience during the pandemic**

1. Your gender is: ________________

2. Your teaching major is: ________________

3. Your school type is: ________________

4. The geographic location of your school is: ________________

5. During the pandemic, did you feel panic and anxious? (Example: media coverage of the new coronavirus)

6. During the pandemic, were you worried about the health of your family and yourself?

7. Did emergency remote teaching (ERT) bring you more challenges and workload?

8. During ERT, were you worried and anxious about the evaluation of teaching performance?

9. Were you afraid of ERT because of your lack of online teaching experience?

10. During ERT, which of the following measures did you take to reduce your psychological burden or anxiety?

a. comfort myself

b. seek help from friends, schools and mental institutions

c. seek help from family

d. distract attention

e. Others: _______________

11. During ERT, do you think the Internet, application software, or teaching management platform brought you challenges?

12. During ERT, do you think the interaction between teachers and students brought you challenges?

13. Did your office location or electronic equipment make it difficult for you to teach online?

14. Did you feel physical discomfort during online classes?

15. During ERT, which of the following measures did you take to solve the difficulties caused by online teaching?

a. universities provide the platform and expenditure

b. simplify the online course process

c. talk with students more frequently

d. familiarize with the online teaching process in advance

e. Others: _______________

16. Were you worried that students would have depression, paranoia, stress and other negative emotions during online teaching?

17. Do you think the pandemic would affect the graduation or work situation of students?

18. Were you concerned about your students’ class attendance during ERT?

19. During ERT, were you concerned about students’ use of technology operations?

20. During ERT, were you concerned that students will not have a suitable location/electronic device to attend class?

21. During ERT, were you worried that students’ lack of online learning experience will reduce their learning efficiency?

22. Were you worried about the physical condition of your students during online teaching?

23. During ERT, which of the following measures did you take to solve students’ psychological burden or anxiety?

a. give comfort to students

b. guide students to relax their mentality

c. provide technical support to students

d. guide students to reduce the burdens

e. Others: _______________

24. Did you encounter challenges from school or departmental systems while teaching online? (For example, the relevant regulations cannot be implemented in a timely and effective manner)

25. During ERT, did you experience challenges due to lack of peer/expert support (e.g., no peer model or technologist for help)

26. During ERT, did you encounter challenges from the surrounding environment (such as unstable community network, frequent nucleic acid testing, etc.)

27. Did you encounter challenges with your child’s learning during online teaching? (For example, children need to be accompanied by parents, time conflicts, etc.)

28. During ERT, which of the following measures did you take to solve the challenges caused by the surrounding environment?

29. Would you like to be the interviewee for this research? If you like, please let us know your WeChat or phone numbers: __________________

Thank you very much! Stay safe during the pandemic!
